# Modifications of *Xanthomonas axonopodis* pv. citri Lipopolysaccharide Affect the Basal Response and the Virulence Process during Citrus Canker

**DOI:** 10.1371/journal.pone.0040051

**Published:** 2012-07-06

**Authors:** Silvana Petrocelli, María Laura Tondo, Lucas D. Daurelio, Elena G. Orellano

**Affiliations:** Molecular Biology Division, Instituto de Biología Molecular y Celular de Rosario, Consejo Nacional de Investigaciones Científicas y Técnicas, Facultad de Ciencias Bioquímicas y Farmacéuticas, Universidad Nacional de Rosario, Suipacha 531, Rosario, Argentina; Universidad de Costa Rica, Costa Rica

## Abstract

*Xanthomonas axonopodis* pv. citri (Xac) is the phytopathogen responsible for citrus canker, one of the most devastating citrus diseases in the world. A broad range of pathogens is recognized by plants through so-called pathogen-associated molecular patterns (PAMPs), which are highly conserved fragments of pathogenic molecules. In plant pathogenic bacteria, lipopolisaccharyde (LPS) is considered a virulence factor and it is being recognized as a PAMP. The study of the participation of Xac LPS in citrus canker establishment could help to understand the molecular bases of this disease. In the present work we investigated the role of Xac LPS in bacterial virulence and in basal defense during the interaction with host and non host plants. We analyzed physiological features of Xac mutants in LPS biosynthesis genes (*wzt* and *rfb30*3) and the effect of these mutations on the interaction with orange and tobacco plants. Xac mutants showed an increased sensitivity to external stresses and differences in bacterial motilities, *in vivo* and *in vitro* adhesion and biofilm formation. Changes in the expression levels of the LPS biosynthesis genes were observed in a medium that mimics the plant environment. Xac*wzt* exhibited reduced virulence in host plants compared to Xac wild-type and Xac*rfb303*. However, both mutant strains produced a lower increase in the expression levels of host plant defense-related genes respect to the parental strain. In addition, Xac LPS mutants were not able to generate HR during the incompatible interaction with tobacco plants. Our findings indicate that the structural modifications of Xac LPS impinge on other physiological attributes and lead to a reduction in bacterial virulence. On the other hand, Xac LPS has a role in the activation of basal defense in host and non host plants.

## Introduction

*Xanthomonas axonopodis* pv. citri (Xac) is the bacterium responsible of citrus canker. Bacteria enter through stomata and wounds in host plants and the disease is visualized as humid circular spots in the abaxial surface of leaves [1]. Later, Xac colonizes the apoplast producing cell hyperplasia and the disease is established as necrotic corky lesions in leaves, fruits and stems [2]. This worldwide disease produces a decrease in quality and quantity of citrus fruits [2], [3].

Lipopolysaccharides (LPSs) are essential and distinctive structures of Gram negative bacteria being a major component of the bacterial cell surface. In general, LPS molecules consist of a hydrophilic heteropolysaccharide formed by three major substructures, the O-specific polysaccharide (O-antigen), composed of a repetitive sugar subunit; the core oligosaccharide region that is covalently linked to the glycolipid moiety lipid A; and the lipid A anchored to the outer side of the plasmatic external membrane [4], [5].

The bacterial LPS molecule confers protection against different environmental stresses, including the hostile medium found inside plant tissues. In this context, the LPS has been recognized as a virulence factor during plant-pathogen interactions [6]. On the other hand, like other components of the bacterial surface such as flagellin, this molecule is capable to induce the basal response in plants acting as a pathogen-associated molecular pattern (PAMP) [7]–[10]. PAMPs have been widely described in bacteria and they can trigger innate defense responses in eukaryotes (plants and animals), being also important for bacterial growth, viability and for the virulence process [11].

One of the most widely studied effects of LPSs on plant cells is their ability to prevent the hypersensitive response (HR) induced in plants by avirulent bacteria. HR is a rapid and localized response characterized by reactive oxygen species (ROS) production and programmed cell death that is often associated with plant host resistance [12].

*Xanthomonas* spp. strains mutant in LPS biosynthesis frequently show reduced virulence with a rapid declining in viable bacterial numbers inside plant tissues. Furthermore, since defective LPSs can no longer protect the cell against aggressive environments, such mutants are often more sensitive to ROS, antibiotics, detergents and antimicrobial peptides [13]–[15]. In addition, the LPS from Xac has been recently implicated in biofilm formation [14], [16].

The genes involved in LPS biosynthesis were identified and characterized by *in silico* analysis in several *Xanthomonas* spp. [17]. In Xac the *wzt* gene (XAC3600) is included in the LPS cluster flanked by *metB* and *etfA*. This gene codes for an ATP-binding protein of an ABC transporter system, involved in the O-antigen biosynthesis. On the other hand, a gene coding for a glycosyltransferase of the LPS core region, *rfb303* (XAC2294), was identified outside this cluster [5], [18], [19].

In a previous report we have determined the structure of purified LPSs obtained from Xac wild-type and a mutant in the *wzt* gene (Xac*wzt*). We have observed striking differences from other *Xanthomonas* LPSs structures described before. Moreover, we have also examined the function of the LPS from Xac in the pathogenesis process suggesting a functional role of the O-antigen moiety in the basal defense response of plants [20].

In this work we investigated the role of *wzt* and *rfb303* genes from Xac during the host and non host plant-pathogen interactions. For that purpose we have constructed an additional Xac mutant in the LPS biosynthesis, specifically in the *rfb303* gene (Xac*rfb303*). We also included in this study the Xac*wzt* strain, previously described. Our results suggest that LPS from Xac presents a dual role during the pathogenesis process acting as a PAMP in the activation of basal defenses and as a virulence factor in the establishment of the citrus canker disease.

## Materials and Methods

### Bacterial Strains, Culture Conditions and Media

*Escherichia coli* cells were cultivated at 37°C in Luria Bertani medium. *X. axonopodis* pv. citri (Xac) cells were grown at 28°C in Silva Buddenhagen (SB) medium [21]. For the *in vitro* studies of pathogen responses to plant-like media, cells were grown in the *hrp*-inducing minimal medium XVM2 [22]. Antibiotics were used at the following final concentrations: ampicillin, 100 µg ml^−1^ for *E. coli* and 25 µg ml^−1^ for Xac; kanamycin, 40 µg ml^-1^; gentamicin, 40 µg ml^−1^. All Xac strains were derivatives of the strain Xcc99-1330 kindly provided by Blanca I. Canteros.

### DNA Manipulation and PCR

All DNA manipulations including plasmid purification, restriction enzyme digestion, DNA ligation and agarose gel electrophoresis were performed with standard techniques [23] unless otherwise specified. Total genomic DNA was isolated by the cetyltrimethylammonium bromide method [24]. Primers used for PCR analysis of *wzt* and *rfb303* genes are listed in Table 1. Genomic DNA (50 ng) was used as the template in a 25-µl reaction mixture. PCR reactions were carried out using Go Taq DNA Polymerase (Promega, USA) in an Eppendorf thermal cycler, with denaturation at 94°C for 5 min and subjected to 30 cycles of denaturation at 94°C for 1 min, annealing at 59°C for 1 min and extension at 72°C for 1.5 min, followed by an incubation at 72°C for 5 min. PCR amplified products were analyzed in 1% (w v^−1^) agarose gels.

**Table 1:** Primers used in this work.

Primer name	Sequence^a^	Amplified fragment
**Primers of ** ***Xanthomonas axonopodis*** ** pv. citri**		
wzt-F	5′ atgcaagcttCCTCTCAAGCGTCTATTCTCGT 3′	
wzt-R	5′ cgcggatccAGGCCCTTATCGGTAAAAAGAC 3′	1031 bp of the XAC3600 gene
wzt-RII	5′ TGAATGGCGACGGAAAAAGC 3′	438 pb of the XAC3600 gene
rfb303-F	5′ atgcaagcttGACATCTCGGCTGACGGTCT 3′	
rfb303-R	5′ cgcggatccAGAAAATGCCAGATCGCAGTG 3′	744 bp of the XAC2294 gene
rfb303-RII	5′ GGAAGTTGAACGGCACAAAC 3′	372 of the XAC2294 gene
Cwzt-F	5′ taggatccaATGACTAGCACTGCGCTTC 3′	
Cwzt-R	5′ atgagctcTCAACGCCCATGGACACG 3′	1230 bp including XAC3600 gene
Crfb303-F	5′ tataagcttaATGGTGTGGCTGTGGTGC 3′	
Crfb303-R	5′-atggatccTCAGGAATTGCGCAATCC-3′	969 bp including XAC2294 gene
R16S-F	5′ TGGTAGTCCACGCCCTAAACG 3′	
R16S-R	5′ CTGGAAAGTTCCGTGGATGTC 3′	217 pb of the XAC4291 gene
**Primers of ** ***Citrus sinensis*** ** cv. Valencia late**		
GSTF	5′ AACCTACTTGGAAACACACTAGAAGA 3′	
GSTR	5′ GTTCATCAGATATCTTAAGGCTGGTA 3′	285 bp of the orange EST sequence of *GST* gene
PR-1F	5′ AAAGTTGTTCAAACTTTTTGTCCTT 3′	
PR-1R	5′ ACATGATCAATAGTAGGGATGTTAGC 3′	252 bp of the orange EST sequence of *PR-1* gene
PrxAF	5′ AGCCGCTCTCATTTCCTCTA 3′	
PrxAR	5′ TTGATCGAAAACAGCCTCTG 3′	247 bp of the orange EST sequence of *PrxA* gene
MKK4F	5′ GGCACCCTCGATACTTTGTT 3′	
MKK4R	5′ TAATTCCCTCCGTAGGCATC 3′	293 bp of the orange EST sequence of *MKK4* gene
actF	5′ CAGCCATCTCTCATCGGAAT 3′	
actR	5′ CCTGTGGACAATGGATGGAC 5′	329 bp of the orange EST sequence of actin gene

a Capital letters correspond to nucleotides of the Xac genome sequence and small letters to nucleotides added to facilitate cloning.

### Mating and Mutagenesis

The Xac mutant strain Xac*rfb303* was constructed by plasmid integration. The amplified product *rfb303*-744 bp using the primer pair rfb303-F and rfb303-R (Table 1) was cloned into the suicide vector pK18mobGII [25] digested with *Bam*HI and *Hind*III, rendering plasmid pK/rfb303. Plasmid was transferred to the Xac wild-type strain by biparental mating from the broad host-range-mobilizing *E. coli* S17-1 strain [26]. Bacterial mixtures were spotted onto Hybond-C membranes, placed on SB agar and incubated for 48 h at 28°C. Membranes were then washed with 0.9% (w v^−1^) NaCl and bacteria transferred to selective medium. Xac mutant strain was selected by the vector-encoded antibiotic resistance (kanamycin). Inactivation of *rfb303* was confirmed by PCR using a combination of the Crfb303-F primer (Table 1) and the M13 reverse primer of the pK18mobGII.

For Xac*wzt* and Xac*rfb303* complementation, DNA fragments containing the *wzt* or *rfb303* coding regions were amplified using the primer pairs Cwzt-F/R or Crfb303-F/R, respectively (Table 1). The amplified DNA fragments were then cloned into the broad-host-range vector pBBR1MCS-5 [27] digested with *Bam*HI/*Sac*I or *Hind*III/*Bam*HI, respectively. The resulting plasmids were transferred to Xac*wzt* and Xac*rfb303* by conjugation, rendering strains Xac*Cwzt* and Xac*Crfb303*, respectively.

### Isolation and Analysis of Lipopolysaccharides

Bacterial cultures of Xac wild-type, Xac*wzt*, Xac*rfb303*, Xac*Cwzt* and Xac*Crfb303* were grown in liquid SB medium to stationary phase (optical density at 600 nm (OD_600_)∼3) and centrifuged for 20 min at 10000 *g*. LPSs from harvested cells were extracted with a 50% phenol-water mixture [28]. The aqueous phases after three extractions were pooled and exhaustively dialyzed (membrane cutoff, 12 kDa) against distilled water at 4°C. LPS solutions were stored at −20°C.

### Analysis of Lipopolysaccharides by Polyacrylamide Gel Electrophoresis

LPS preparations were solubilized in sample buffer 3× (0.187 M Tris-HCl (pH 6.8), 6% (w v^−1^) sodium dodecyl sulfate (SDS), 30% (v v^−1^) glycerol, 0.03% (w v^−1^) Bromophenol Blue, 15% (v v^−1^) 2-mercaptoethanol) and visualized in a 3% (w v^−1^) stacking gel and a 14% (w v^−1^) separation gel using the tricine-SDS polyacrylamide gel electrophoresis (SDS-PAGE) system described by Marolda *et al.* [29].

Samples (30 µl) were loaded and run in a Mini-Protean III vertical electrophoresis cell (Bio-Rad) at 50 volts until the dye reached the resolving gel (30–40 min) and then switched to 130 volts and run for additional 20–30 min after the dye left the gel. The gel was incubated in fixing solution I (50% (v v^−1^) methanol, 12% (v v^−1^) acetic acid) overnight before silver stained by a method adapted from Tsai and Frasch [30]. After three rinses in 95% (v v^−1^) ethanol, the gel was incubated during 15 min with freshly made fixing solution II (40% (v v^−1^) methanol, 0.05% (v v^−1^) formaldehyde). The gel was washed twice with milli-Q deionized water, the LPSs were oxidized in the gel with 0.2% (w v^−1^) periodic acid for 30 min and the gel was rinsed in milli-Q deionized water. The staining solution was made up immediately before using by slowly adding 2.5 ml of 20% (w v^−1^) silver nitrate solution to 14 ml of 0.1 M NaOH containing 1 ml of aqueous ammonia. This solution was shaken to dissolve the brown precipitate and diluted with 57.5 ml of milli-Q deionized water. After rocking in the staining solution for 10 min, the gel was washed 6 times with milli-Q deionized water. Sodium carbonate (0.28 M) containing 0.5 ml of formaldehyde and 0.1 ml of 1% (w v^−1^) sodium thiosulfate per liter was added to develop the ladder bands, and the reaction was stopped with fixing solution I.

### Survival in the Presence of Hydrogen Peroxide

Survival experiments were performed by subculturing Xac wild-type, Xac*wzt*, Xac*rfb303* and the complemented strains over night cultures into fresh SB medium at 2% inoculums. After 6 h of growth (early exponential phase) aliquots of the cultures were diluted and plated on SB-agar plates. Hydrogen peroxide was then added to the cultures at final concentrations of 0.5 and 1 mM. After 15 min of exposure to the oxidant, samples were removed, washed once with fresh medium, serially diluted and plated on SB-agar plates. Colonies were counted after 48 h incubation at 28°C. The percentage of survival was defined as the number of colony-forming units (CFU) after treatment divided by the number of CFU prior to treatment ×100.

### Sensitivity to SDS and MV

The sensitivity of Xac wild-type, the mutant and the complemented strains to SDS was assessed by growing the bacteria in SB-1.5% (w v^−1^) agar plates containing 0.01 and 0.1% (w v^−1^) SDS. Plates were incubated at 28°C during 48 h.

Bacterial resistance to MV was evaluated by the disk diffusion method. Briefly, 100 µl of a bacterial suspension (containing ∼10^9^ cells ml^−1^) was mixed with 3 ml of SB-0.7% (w v^−1^) molten agar and was poured onto SB-agar plates supplemented with the corresponding antibiotics. After hardening, 5 µl of a 50, 100 or 500 mM MV solution was added onto paper disks (5-mm diameter) placed on the agar surface. The zones of growth inhibition were measured after incubation for 48 h at 28°C.

### Bacterial Motility Assays

For swimming and swarming assays, over night cultures of Xac wild-type, the mutant and the complemented strains were subcultured into fresh SB medium at 2% inoculums and grown to late exponential phase (15 h). Bacteria were harvested by centrifugation and resuspended in fresh SB medium adjusting the bacterial concentration to 10^7^ CFU ml^−1^. SB plates with 0.3 and 0.7% (w v^−1^) agar respectively were inoculated with 3 µl of the corresponding bacterial suspension and incubated for 4 days at 28°C [31].

### Western Blot Analysis

Flagellin levels were determined by Western Blot analysis using polyclonal anti-flagellin rabbit antibodies from *Serratia marcesens*, kindly provided by Dr. Eleonora García Véscovi. Bacteria from the border and the center regions of the migration zones of swarming plates were collected and resuspended in 500 µl of PBS buffer (8 g l^−1^ NaCl, 1.15 g l^−1^ Na_2_HPO_4_·7H_2_O, 0.2 g l^−1^ KH_2_PO_4_, pH 7.4). Protein extraction and Western Blot analyses were performed as described by Sambrook *et al*. [23].

### Quantification of Exopolysaccharide (EPS) Production

For the EPS quantification, Xac strains were grown in liquid SB medium at 28°C for 5 days. Bacteria were harvested by centrifugation and EPS was precipitated from the culture supernatant by the addition of two volumes of ethanol. The precipitate was vaccum filtrated and weighed [32].

### Biofilm Formation Assay

For the analysis of biofilm formation, Xac strains were transformed by biparental mating with plasmid pBBR1MCS-2EGFP expressing the green fluorescent protein (GFP) [33]. *E. coli* S17-1 cells harboring this plasmid were conjugated to the different Xac strains and transconjugants were selected for gentamicin resistance.

Overnight cultures of the GFP-labeled strains in SB medium were adjusted to the same OD_600_, diluted 1∶100 in fresh medium and 300 µl placed into chamber covered glass slides (N°155411, Lab-Tek, NUNC, Naperville. IL, U.S.A.). Chambers were statically incubated in a humidified polyvinylchloride (PVC)-box at 28°C. The biofilm formation was visualized by confocal laser scanning microscopy (CLSM) (Nikon Eclipse TE-2000-E2) with motor system and DIC/Nomarski optics and a head scan D Eclipse C1si. The images obtained were analyzed with Nikon EZ-C1 3.90 software.

### Plant Material and Plant Inoculations

Orange (*Citrus sinensis* cv. Valencia late) was used as host plant and tobacco (*Nicotiana tabacum* cv. Petit Havana) as non host plant for Xac. All plants were grown in a growth chamber in incandescent light at 25°C with a photoperiod of 16 h. Overnight cultures of Xac strains were diluted in 10 mM MgCl_2_ to a final concentration of 10^7^ CFU ml^−1^. Bacterial suspensions were infiltrated into the intercellular spaces of fully expanded leaves with needleless syringes. *In planta* growth assays were performed by grinding 0.8-cm-diameter leaf discs from infiltrated leaves in 100 µl of 10 mM MgCl_2_, followed by serial dilutions and plating onto SB-agar plates supplemented with the appropriate antibiotics. Colonies were counted after 48 h incubation at 28°C [21].

### Ion Leakage

For ion leakage determination, one week after the infiltration of leaves with the different bacterial strains, 0.8-cm-diameter leaf discs were collected from the inoculated areas and washed with water for 30 min. Discs were then placed in microtubes with 1 ml of distilled water and incubated for 4 h before conductivity measurements were performed. Samples were then destroyed by autoclaving and allowed to leak for an additional period of 2 h. The conductance of boiled leaf discs was taken as 100% ion content [21].

### Bacterial Adhesion to Abiotic and Biotic Surfaces

To measure the level of cell adherence to a plastic surface, bacterial cultures of Xac wild-type, Xac*wzt*, Xac*rfb303*, Xac*Cwzt* and Xac*Crfb303* were grown overnight in liquid SB and XVM2 media. Cells from 1 ml of culture were harvested by centrifugation, washed and resuspended in the same growth medium. Then, 100 µl of each bacterial suspension and the negative controls (growth media) were inoculated into each well of 96-well PVC microtiter plates, incubated for 6 h at 28°C and stained by adding 25 µl of a 1% (w v^−1^) solution of crystal violet (CV) to each well. The plates were incubated at room temperature for 15 min and bacterial adhesion was measured after rinsed the plates with water to remove non-adherent cells. The CV dye was solubilized by the addition of 200 µl of 95% (v v^−1^) ethanol to each well and then quantified by measuring absorbance at 540 nm.

To analyze bacterial adherence to leaf surfaces, 20 µl of each bacterial suspension were inoculated on the abaxial face of orange leaves and incubated for 6 h at 28°C in a humidified chamber. Then, leaves were stained with a solution of 0.1% (w v^−1^) CV at room temperature for 15 min and rinsed with water to remove non-adherent cells. Discs of the inoculated areas were ground and transferred to microtubes, and the dye was quantified as previously described [34].

### RNA Preparation and Gene Expression Analysis

Plant and bacterial total RNA was isolated using TRIzol® reagent (Invitrogen) according to the manufacturer’s recommendations. After extraction, the RNA was treated with RNase-free DNase (Promega) and its integrity was checked by agarose gel electrophoresis. cDNA first strand was synthesized from 1 µg of total RNA as template using 200 U M-MLV Reverse Transcriptase (Promega, USA), 0.5 mM dNTP mixture, 2.5 µg oligonucleotide dT_22_ for plant RNA or 0.5 µg gene-specific primers for bacterial RNA (wzt-RII or rfb303-RII, see Table 1), and incubating for 60 min at 42°C. Control reactions, where retrotranscription was omitted, were done in parallel for all the samples to rule out the possibility of amplification from contaminating DNA.

For bacterial gene expression PCR reactions were carried out with 2 µl cDNA template under the following conditions: 25 cycles of denaturation at 94°C for 1 min, annealing at 59°C for 1 min, and extension at 72°C for 1 min; with a final extension step at 72°C for 5 min. The number of cycles to be used, avoiding reaching the plateau of the PCRs, was previously determined by taking samples at different number of cycles during the PCR amplification step and analyzing the products obtained by agarose gel electrophoresis. As a constitutive control, a 217-bp fragment of the bacterial 16S rRNA was amplified using the same PCR conditions. RT-PCR products were resolved on 1.5% (w v^−1^) agarose gels, and densitometrically quantified using Gel-Pro Analyzer Software 3.1 (Media Cybernetics).

For the analysis of plant gene expression real-time PCR was performed with a Realplex Instrument (Eppendorf) equipped with Realplex Software version 4.0. Reactions were performed with 1 µl cDNA template and a homemade SYBRgreen-I reaction mixture [35], containing 1∶50000 diluted SYBR green-I (Invitrogen), 10 pmol of gene specific primers (Table 1), 0.5 U Platinum-Taq DNA polymerase (Invitrogen), 40 nmol dNTP mixture, 3.75 mM MgCl_2_ and 1× Platinum-Taq buffer in a final volume of 20 µl under the following conditions: 95°C for 1 min followed by 40 cycles of 95°C for 15 s, 59°C for 20 s and 72°C for 40 s. Fluorescent intensity data were acquired during the 72°C extension step. Specificity of the amplification reactions was assessed by agarose gel electrophoresis and melting curve analyses, which were run at 95°C for 15 s and 60°C for 15 s followed by an increase in temperature from 60 to 85°C (0.2°C s^−1^) with continuous fluorescence recording. To perform the analysis of relative expression we used the 2^−ΔΔCT^ method [36], normalizing to actin expression levels. All real-time PCR experiments were performed in duplicate.

## Results

### Isolation and Analysis of LPSs from Xac Mutants in LPS Biosynthesis

To assess the involvement of *rfb303* gene in Xac LPS biosynthesis a mutant strain, Xac*rfb303*, was constructed and genetically verified by PCR analysis (data not shown).

**Figure 1:**
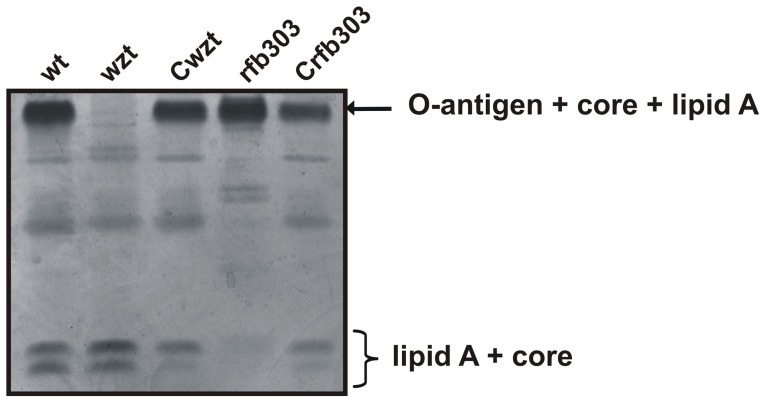
Analysis of Xac LPSs by SDS-PAGE LPSs were isolated from Xac wild-type (lane 1), Xac*wzt* (lane 2), Xac*Cwzt* (lane 3), Xac*rfb303* (lane 4) and Xac*Crfb303* (lane 5) strains by the hot phenol method. The polyacrylamide gel was run with a tricine buffer system and subsequently silver-stained.

LPSs from Xac wild-type, Xac*wzt* and Xac*rfb303* [20], were purified by the hot phenol method [28] and analyzed by SDS-PAGE (Figure 1). As was previously described, the LPS from Xac wild-type showed one well defined slow migrating band that corresponds to the entire LPS, composed of the O-antigen + core + lipid A moiety, and two faster migrating bands. The upper band corresponds to lipid A + core and the lower band to lipid A + inner core [20]. The Xac*wzt* LPS lacked the slow migrating band, corresponding to the complete LPS molecule containing a polymeric O-antigen, when compared with Xac wild-type LPS. The electrophoretic mobility of the rapid migrating bands representing the lipid A + core structure were not influenced in this mutant [20]. The analysis of LPS from Xac*rfb303* showed a similar band pattern to Xac wild-type, although some bands of intermediate migration were detected. These bands could represent intermediates of the LPS biosynthesis consisting of different amounts of the core or oligosaccharides subunits or truncated lipid A + core moieties [37], [38]. LPSs isolated from Xac*Cwzt* and Xac*Crfb303* complemented strains recovered the phenotype observed for LPS from Xac wild-type (Figure 1).

### Sensitivity of Xac*wzt* and Xac*rfb303* to Oxidative Stress and SDS

**Figure 2:**
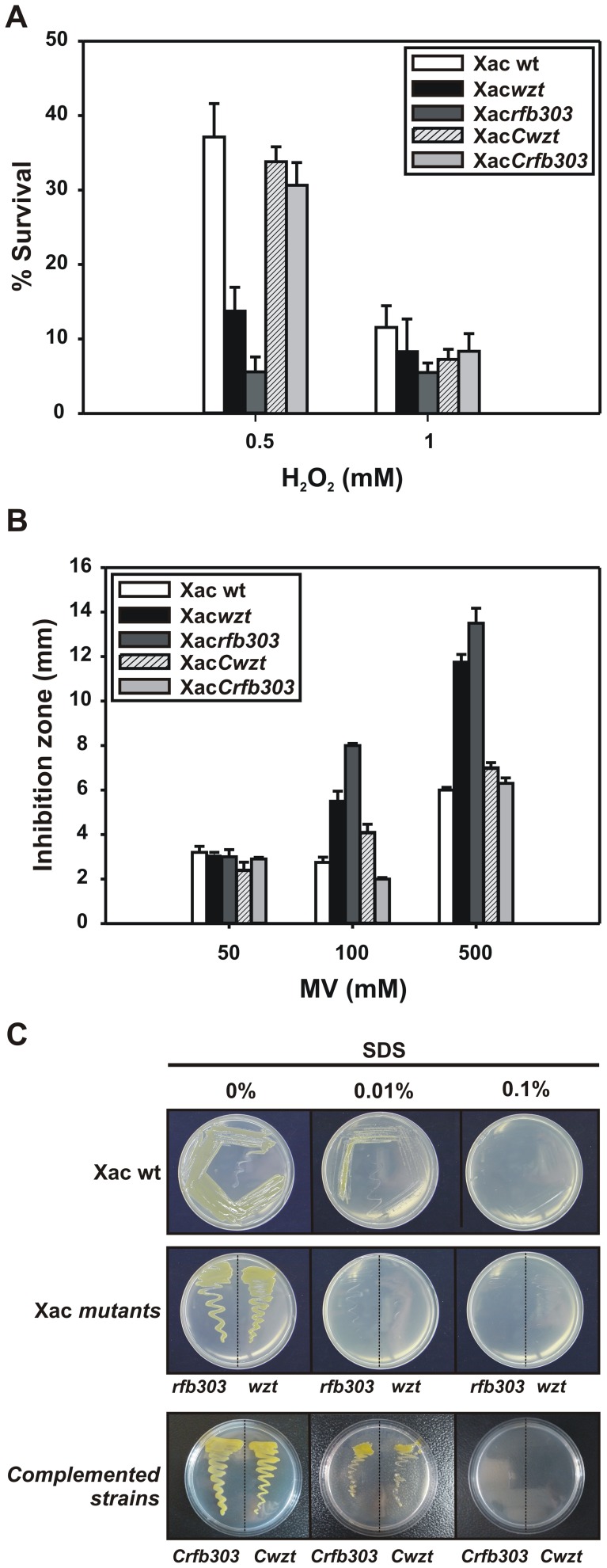
Sensitivity of Xac wild-type, Xac*wzt* and Xac*rfb303* to oxidative stress and SDS (A) Hydrogen peroxide resistance of bacterial cultures. Cells in early exponential phase of growth were exposed to the indicated concentrations of H_2_O_2_ for 15 min. The number of CFU was determined for each culture before and after the peroxide treatment by plating of appropriate dilutions. The percentage of survival is defined as the number of CFU after treatment divided by the number of CFU prior to treatment ×100. (B) Susceptibility to MV toxicity by the disk diffusion assay. The diameters of the inhibition zones were measured after 24 h of incubation. In (A) and (B) data are expressed as the mean ± standard deviation of three independent experiments. (C) Growth of Xac strains in SB-agar plates supplemented with different concentrations of SDS.

Because the outer membrane is considered a permeability barrier for harmful substances, we tested the sensitivity of Xac*wzt* and Xac*rfb303* strains to oxidant compounds (hydrogen peroxide and MV) and SDS, an anionic detergent that usually affects membrane integrity. Both mutants exhibited lower survival rates after 0.5 mM hydrogen peroxide treatment and larger zones of growth inhibition in MV-containing plates, compared to the wild-type strain (Figure 2A and 2B). In addition, the mutant strains failed to grow in the presence of low concentrations of SDS (Figure 2C). The complemented strains Xac*Cwzt* and Xac*Crfb303* reverted to Xac wild-type sensitivity to hydrogen peroxide, MV and SDS (Figure 2).

### Impact of *wzt* or *rfb303* Mutation on Bacterial Motility, Adhesion and Biofilm Formation

**Figure 3:**
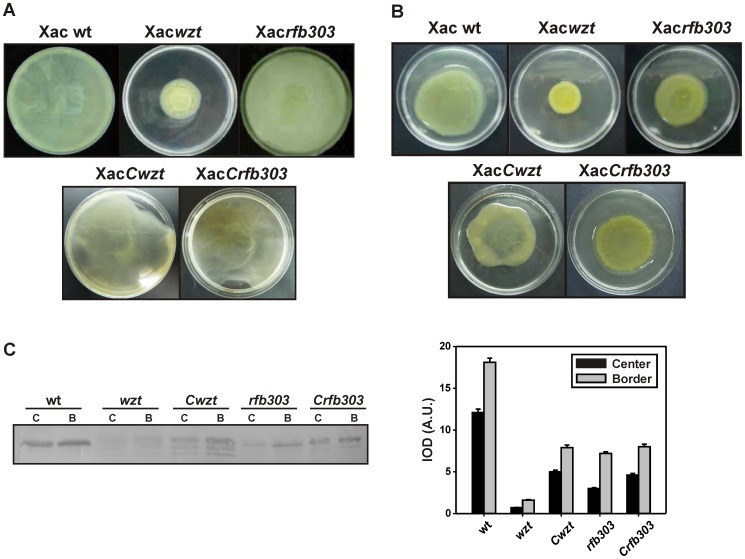
Bacterial motility assays. The different strains were centrally inoculated on SB plates supplemented with 0.3% (w v^−1^) agar for swimming (A) and 0.7% (w v^−1^) agar for swarming assay (B) and incubated 4 days at 28°C to determine migration zones. (C) Analysis of flagellin expression of bacteria from the border and the center of swarming plates by Western Blot using *Serratia marcescens* anti-flagellin rabbit polyclonal antibodies (left panel). Expression profiles were obtained by densitometric quantification of band intensities (right panel). Experiments were performed in triplicate with similar results; bars indicate mean ± standard deviation. IOD, integrated optical density; A.U., arbitrary units; C: center and B: border zone of a swarming plate.

Bacteria use a variety of motility mechanisms to colonize environments such as nutrient-rich surfaces and host tissues. These motilities include flagellum-dependent swimming and swarming [31]. Xac is a motile bacterium with a single polar flagellum able to slide on liquid medium by swimming and on semi-solid agar by swarming [3]. We evaluated if LPS biosynthesis mutations of Xac affected bacterial motility. Xac*wzt* displayed strongly diminished swimming capacity on SB-0.3% (w v^−1^) agar plates. However, no differences were observed between Xac*rfb303* and Xac wild-type swimming motility (Figure 3A). When swarming was assessed on SB-0.7% (w v^−1^) agar plates higher migration diameters were observed for Xac wild-type and Xac*rfb303* respect to Xac*wzt*. Moreover, Xac wild-type motilities were restored in the complemented strains (Figure 3A and 3B).

The expression of flagellin was then analyzed by Western Blot in cells of Xac wild-type and mutant strains obtained from the border and the center regions of swarming plates (Figure 3C). A simultaneously run Coomassie-stained gel indicated equal protein loadings between samples (Figure S1). As expected, expression of flagellar protein in wild-type bacteria increased from the center to the border of the migration zone. A similar behavior was also observed in Xac*rfb303* cells, but lower levels of flagellin were observed for this strain. In contrast, Xac*wzt* flagellin contents were barely detectable compared to levels observed in Xac wild-type. Xac*Cwzt* and Xac*Crfb303* showed the same behavior than wild-type cells, but comparable flagellin levels to Xac*rfb303* mutant (Figure 3C).

**Figure 4:**
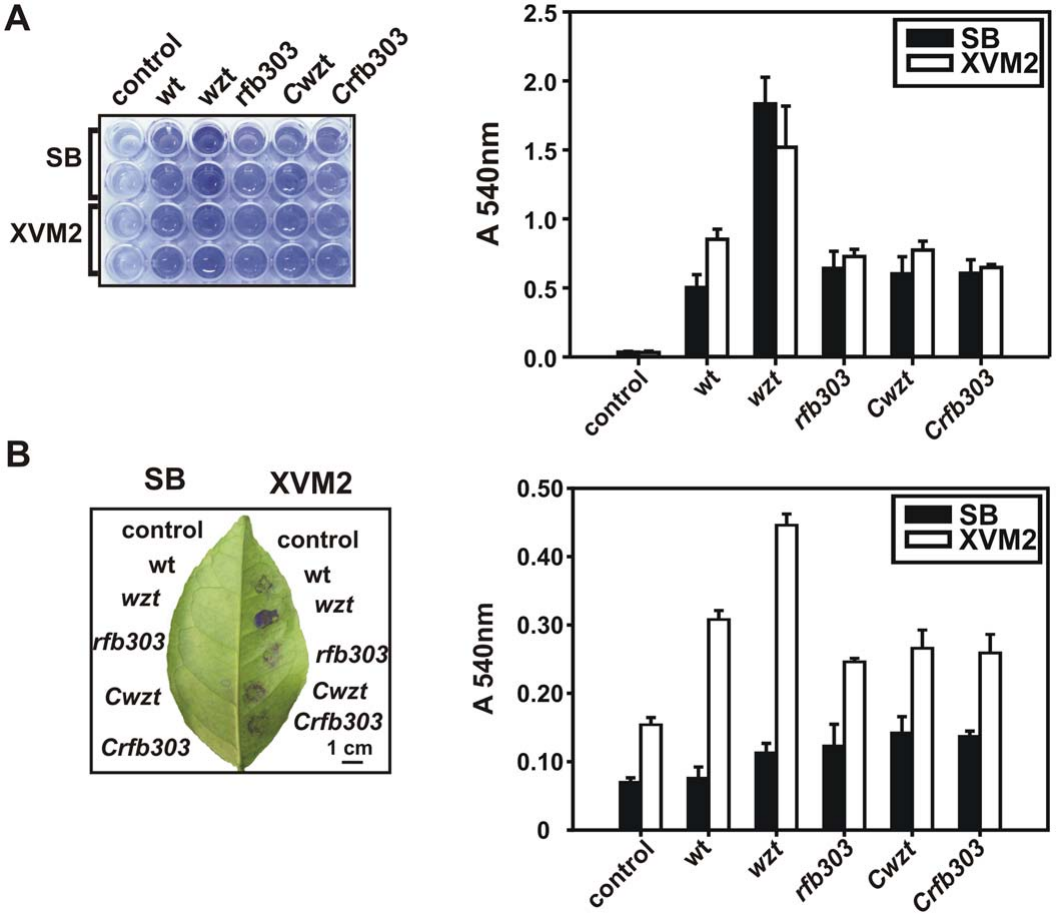
Bacterial adhesion to abiotic and biotic surfaces. (A) Bacterial adhesion on plastic (PVC microtiter plate) surface of Xac wild-type, Xac*wzt*, Xac*rfb303*, Xac*Cwzt* and Xac*Crfb303* strains grown in SB or XVM2 medium. (B) Bacterial adhesion on abaxial orange leaves surfaces. In the left, representative images of CV staining of a PVC plate or a leaf are shown. Histograms in the right represents spectrophotometric quantifications of CV attached (Abs 540 nm). Data are expressed as the mean ± standard deviation of three independent experiments. Scale bar, 1 cm.

To determine if Xac LPS is involved in bacterial adhesion we analyzed Xac wild-type, Xac*wzt* and Xacr*fb303* adherence ability to abiotic and biotic surfaces. *In vitro* assays were performed by incubating SB or XVM2 grown cultures in PVC microtiter plates and staining the attached cells with CV (Figure 4A). Solubilization of the CV stain by addition of ethanol provides an indirect, quantitative measurement of the adherent cell mass in a given well. Xac*wzt* showed higher adherence than Xac wild-type and Xac*rfb303* strains to the plastic surface, with no differences between both growth media. On the other hand, Xac wild-type and Xac*rfb303* strains presented more adhered cells when cultures were grown in XVM2.

*In vivo* assays were performed incubating cultures on abaxial orange leaf surfaces. All bacterial strains displayed higher attachment when grown in XVM2 medium. In addition, Xac*wzt* showed more CV staining than Xac wild-type and Xac*rfb303* strains. When cells were grown on SB medium similar levels of CV staining were observed between the bacterial strains and the controls, indicating absence of adherence. *In vitro* and *in vivo* adhesion capabilities of the complemented strains were similar to Xac wild-type when the bacteria were grown in SB and in XVM2 media (Figure 4B).

**Figure 5:**
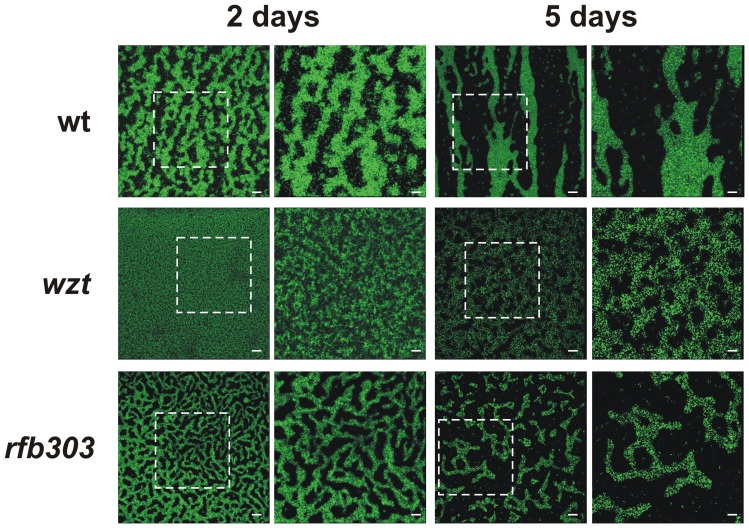
Biofilm formation. GFP-labeled Xac strains were grown on chambered cover slides and visualized under CLS microscopy after 2 and 5 days of bacterial growth. For each time period the left panels show cell aggregation at the bottom of the chambered cover slides with a magnification of 40× and the right panels show a 2× zoom of the regions marked in the previous panels. Scale bars, 50 µm.

We analyzed structural characteristics of bacterial biofilms developed by GFP-labeled strains of Xac on chambered cover glass slides over different periods of time, using CLSM (Figure 5). Xac wild-type and Xac*rfb303* developed a structured biofilm with microcolonies and clustered bacteria in close contact with each other at 2 days of incubation. On the other hand, a flat lawn of bacteria without any organized structure was observed in static cultures of Xac*wzt*. By 5 days of culture more complex structures and different patterns of bacterial aggregation between Xac wild-type and Xac*rfb303* were observed. Xac*rfb303* presented more densely packed and smaller microcolonies whereas Xac wild-type generated large aggregates that were extended over the entire surface. Moreover, Xac*wzt* generated small structures that were considerably less organized than those produced by Xac wild-type and Xac*rfb303*. In addition, less production of EPS was observed for Xac*wzt* mutant compared with Xac wild-type and Xac*rfb303* (Table 2). This was consistent with a less mucoid aspect of Xac*wzt* colonies observed on solid growth medium (Figure S2).

**Table 2:** Xanthan production in liquid medium of Xac strains.

Bacterial strain	EPS (mg ml^−1^)^a^
Xac wt	6.6±0.2
Xac*wzt*	3.8±0.6
Xac*rfb303*	4.7±0.3
Xac*Cwzt*	5.6±0.4
Xac*Crfb303*	6.7±0.3

a Data represent mean ± standard deviation of three independent experiments.

### Xac LPS Genes Expression in a Medium that Mimics the Environment of Plant Intercellular Spaces

**Figure 6:**
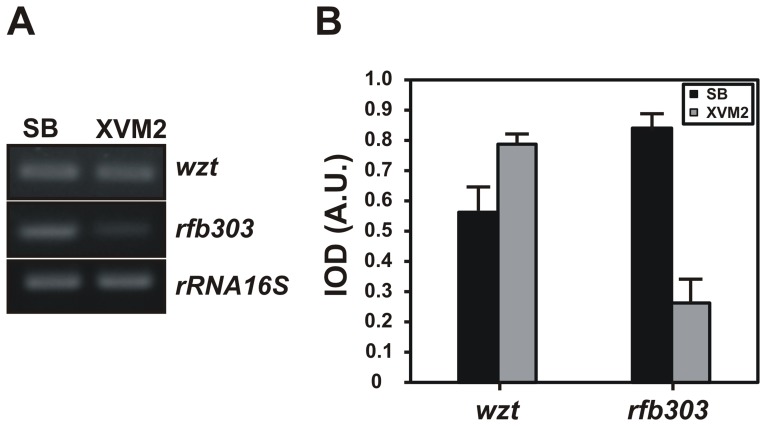
Expression of Xac LPS genes in the plant-mimicking XVM2 medium. (A) Amplified products of the *wzt* and *rfb303* genes by semiquantitative RT-PCR using RNA preparations from early exponential Xac cultures grown in SB and in XVM2. As a control for constitutive bacterial expression a fragment of 16S rRNA was simultaneously amplified. (B) Expression profiles obtained by densitometric quantification of band intensities. Data are expressed as the mean ± standard deviation of three independent experiments. IOD, integrated optical density; A.U., arbitrary units.

We compared the expression levels of *wzt* and *rfb303* genes from Xac in early exponential phase cultures grown in SB, a rich standard medium, and in XVM2, a minimum medium that simulates conditions in the apoplastic space of plants, inducing the bacterial *hrp* (for *h*ypersensitive *r*esponse and *p*athogenicity) gene cluster [22]. Interestingly, while the expression levels of *wzt* were similar in both media, expression of *rfb303* was ∼3.4-fold lower in XVM2 than in SB (Figure 6).

### Disease Development Analysis in Citrus Plants Infected with Xac*wzt* and Xac*rfb303* Mutants

**Figure 7:**
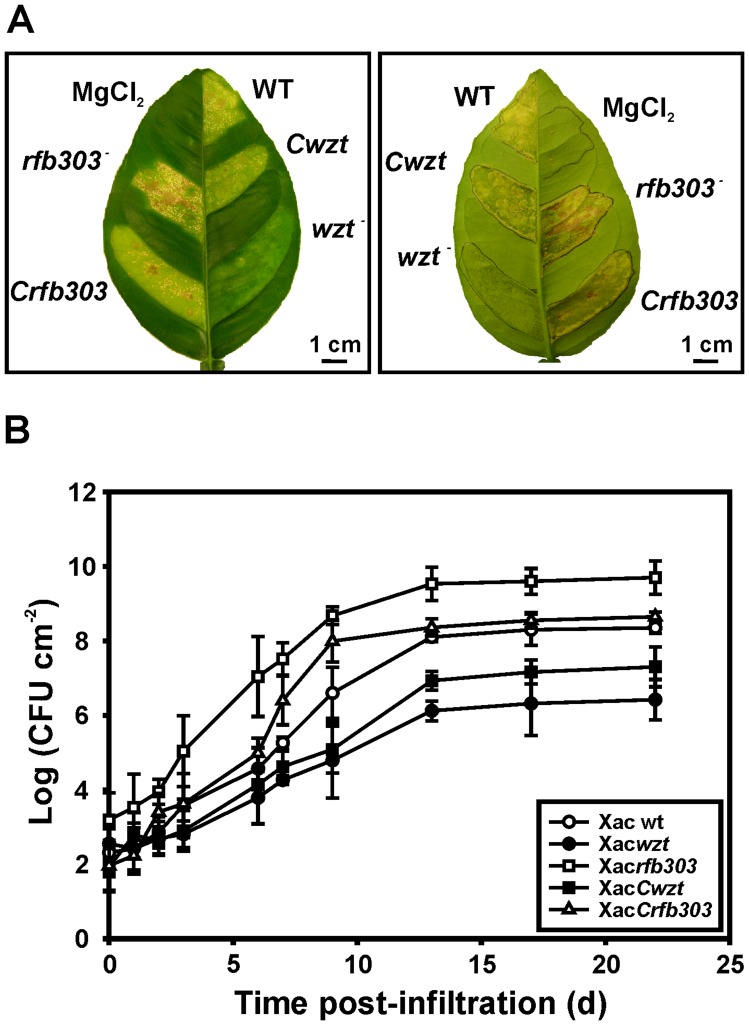
Effect of *wzt* and *rfb303* disruption on pathogenicity in host plants (A) Disease symptoms on orange leaves inoculated with Xac wild-type, the LPS mutants Xac*wzt* and Xac*rfb303* and the complemented strains Xac*Cwzt* and Xac*Crfb303* at 10^7^ CFU ml^−1^ in 10 mM MgCl_2_. A representative leaf 13 days after inoculation is shown. Left panel, adaxial side; right panel, abaxial side. Scale bars, 1 cm. (B) Bacterial growth of Xac cells in orange leaves during 22 days. Values represent means ± standard deviations of three independent samples.

In order to assess the effect of *wzt* and *rfb303* mutations on Xac virulence, the mutant strains were tested for their ability to trigger disease in citrus leaves. The Xac*rfb303* mutant produced typical canker lesions upon infiltration at a concentration of 10^7^ CFU ml^−1^ and a higher percentage of necrotic area than the wild-type strain (Figure 7A). Differences were also observed in the time of appearance of the first symptoms (water soaking), which was relatively shorter in this mutant (data not shown). On the other hand, the magnitude of the lesions and the number of cankers were significantly diminished in the Xac*wzt* mutant compared to wild-type bacteria, even though the infiltration areas and the bacterial densities were equivalent for all the strains. Infiltration with Xac*Cwzt* and Xac*Crfb303* complemented strains caused similar symptoms and a comparable percentage of necrotic area than wild-type cells (Figure 7A).

The degree of virulence of the different strains was also evaluated by conducting bacterial growth curves *in planta*. As shown in figure 7B, the magnitudes of leaf injuries correlated with the bacterial growths inside host tissues. The bacterial number of Xac*rfb303* recovered from the infected leaves at 22 days post-infiltration (dpi) (∼10^9^ CFU cm^−2^) was significantly higher than that of the wild-type strain (∼10^8^ CFU cm^−2^); whereas the bacterial number of the Xac*wzt* mutant was noticeably lower (∼10^6^ CFU cm^−2^). Moreover, although complementation restored the bacteria to virulence on citrus leaves, Xac*Cwzt* growth on leaves did not reach the values of the wild-type (Figure 7B).

Ion leakage, correlated with cell death [39], was measured in order to estimate the degree of cell membrane injury produced by the different bacterial strains in orange plants. Values of 33% ion leakage were obtained for Xac wild-type, relative to 10 mM MgCl_2_ treatment 7 dpi; for Xac*rfb303*, values were of 30%; and for Xac*wzt*, only 7%, indicating less damage in leaves inoculated with this mutant (Table 3).

**Table 3:** Cell membrane injuries of orange and tobacco leaves produced by bacterial infection.

	Ion leakage (%)^a^	
Bacterial strain	Orange	Tobacco
Xac wt	33.00 ± 1.06	56.40 ± 0.73
Xac*wzt*	7.69 ± 0.27	3.30 ± 0.56
Xac*rfb303*	30.68 ± 0.58	3.60 ± 0.35

The leaves were inoculated with Xac wild-type, Xac*wzt* and Xac*rfb303* at 10^7^ CFU ml^-1^ and the assays were performed at 7 (orange) or 2 (tobacco) days post-infiltration.

a Data represent mean ± standard deviation of three independent experiments.

### Expression Analysis of Defense-related Genes in Orange Leaves

**Figure 8:**
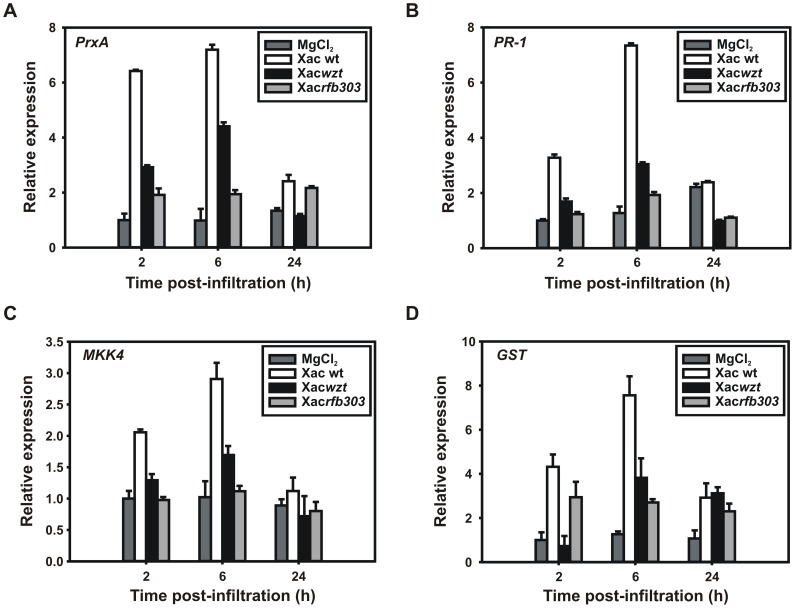
Expression analysis of *C. sinensis* defense-related genes (A) Real Time PCR showing expression levels of *PrxA*, *PR-1*, *MKK4* and *GST* genes from orange leaves infiltrated with Xac wild-type, Xac*wzt* and Xac*rfb303* at 10^7^ CFU ml^−1^ and 10 mM MgCl_2_ as control. Leaves were harvested at 2, 6 and 24 hpi. Columns show the expression value relative to the control. To perform the analysis of relative expression we used the 2^−ΔΔCT^ method normalizing to actin expression levels. The results are expressed as mean ± standard deviation of three independent determinations.

In order to evaluate the response of orange plants to Xac wild-type and LPS mutants we conducted real-time PCR analysis of some well-characterized pathogen-responsive genes. For this purpose the pathogenesis-related protein-1 (PR-1), the signaling protein mitogen-activated kinase kinase 4 (MKK4), the antioxidant enzymes glutathione-S-transferase (GST) and peroxiredoxine A (PrxA) were chosen. As shown in figure 8, transcript levels in response to Xac wild-type exhibited an early accumulation at 2 h post-infiltration (hpi) for all the genes tested, subsequently increasing to reach maximal levels at 6 hpi and decreasing at 24 hpi. The Xac*wzt* mutant caused a relatively weak increase in the accumulation of transcripts at 6 hpi that also decayed at 24 hpi. In contrast, Xac*rfb303* did not induce a substantial change in gene expression, with only a weak increase in the transcript levels of *PrxA* and *GST* at 2 hpi that remained constant to 24 hpi (Figure 8).

### Interaction of Xac*wzt* and Xac*rfb303* with Non Host Plants

**Figure 9:**
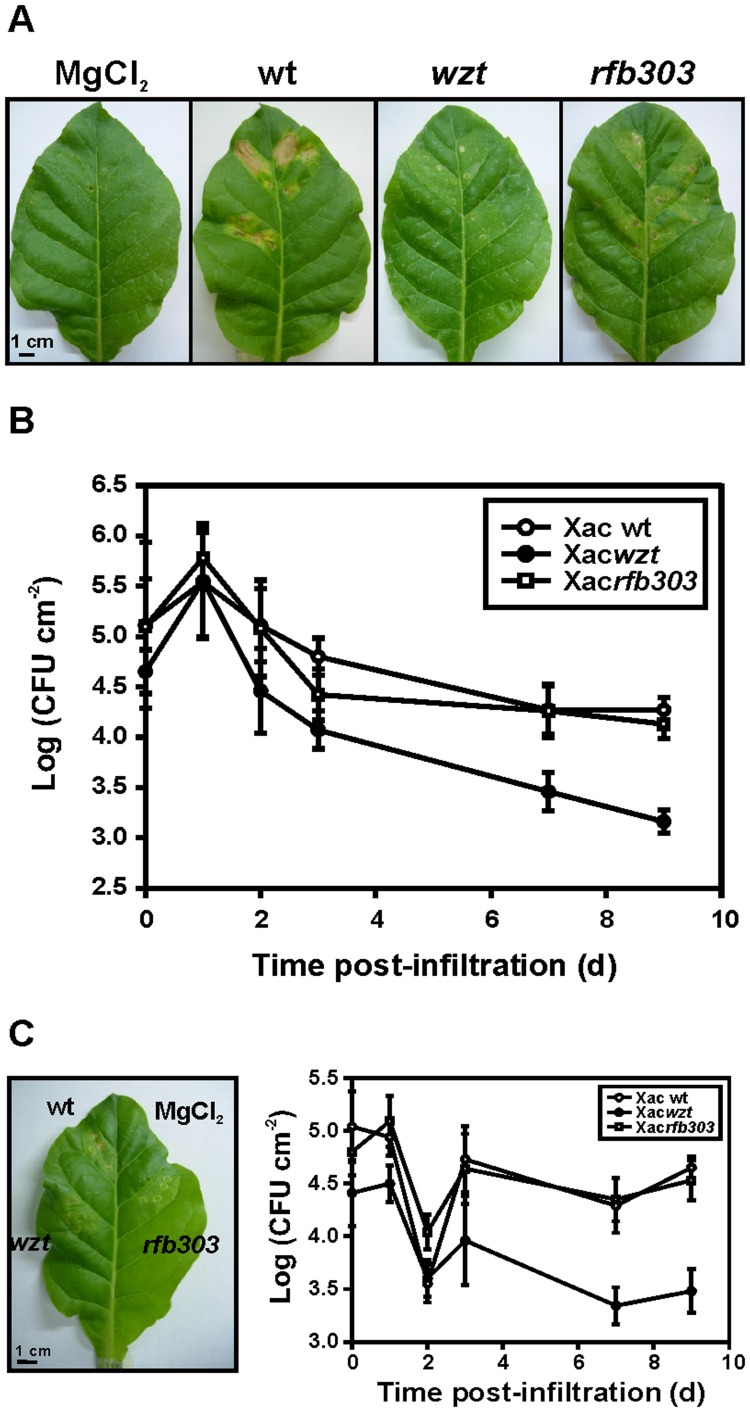
Interaction of Xac strains with non host plants. (A) Phenotype developed on tobacco leaves inoculated with Xac wild-type, Xac*wzt* and Xac*rfb303* at 10^7^ CFU ml^−1^ in 10 mM MgCl_2_. Representative leaves are shown 24 hpi. (B) Bacterial growth of Xac wild-type, Xac*wzt* and Xac*rfb303* in tobacco leaves during 9 days. Values represent the mean ± standard deviation of three independent experiments. (C) Effect of pre-inoculation of tobacco leaves with LPS from Xac wild-type. A tobacco leaf area was inoculated with LPS (100 µg ml^−1^) and 20 h later, Xac strains at 10^7^ CFU ml^–1^ were inoculated into the same area. A representative 24 hpi leaf and bacterial growth curves during 9 days are shown. Scale bars, 1 cm.

To analyze the role of Xac LPS during non host interactions, tobacco leaves were inoculated with Xac wild-type, Xac*wzt* and Xac*rfb303*. HR was visualized at 24 hpi in leaves inoculated with Xac wild-type and the lesion was characterized by a brown and dried necrotic area at the site of infection. In contrast, no HR was generated by inoculation of mutant strains (Figure 9A). Ion leakage was measured to confirm the phenotype observed. For Xac wild-type inoculation, values of 56% ion leakage were obtained, relative to buffer treatment 2 dpi; for Xac*wzt* and Xac*rfb303*, values obtained were markedly lower than Xac wild-type with ∼3% (Table 3). However, *in planta* bacterial growth curves exhibited similar patterns for the three strains (Figure 9B). The number of recovered bacteria showed an increase at 24 hpi and then began to decline at 2 dpi.

One of the most widely studied effects of LPSs on plant cells is their ability to prevent the HR induced by avirulent bacteria [13]. Pre-treatment of tobacco leaves with LPS from Xac wild-type at 100 µg ml^−1^ prevented HR induced by Xac wild-type (Figure 9C). Besides, the initial bacterial growth previously described during the non host interaction was not observed in LPS pre-treated leaves, for both wild-type and mutant strains. In contrast, bacterial number diminished at 2 dpi and then showed a weak increase and remained constant during the 9 days of the assay.

## Discussion

The LPS is an important component of Gram negative bacteria and its role in the animal- and plant-pathogen interactions has been widely studied [40], [41]. In a previous work we have characterized the LPS molecule from *X. axonopodis* pv. citri wild-type and from a mutant in the LPS biosynthesis, Xac*wzt*, demonstrating that the O-antigen moiety induces the innate immunity in orange plants [20]. The Xac*wzt* strain is a mutant in the O-antigen ABC-type transporter encoded by *wzt* gene [17].

In order to determine the role of LPS from *X. axonopodis* pv. citri during citrus canker disease we have constructed another mutant strain in the LPS core region, Xac*rfb303*. The *rfb303* gene encodes a putative LPS core biosynthesis protein, implicated in the glycosylation of this region [18]. To date no reports were published describing a *X. axonopodis* pv. citri mutant with a modification in the LPS core region.

The different LPS species isolated from *X. axonopodis* pv. citri wild-type, Xac*wzt* and Xac*rfb303* were further characterized by SDS-PAGE. The band pattern observed in the LPS from *X. axonopodis* pv. citri wild-type was similar to those of other *Xanthomonas* spp. [37], [38], whereas the LPS recovered from the mutant strains exhibited differences that were consistent with the assigned roles of the mutated genes (Figure 1).

Several reports demonstrated that LPS is important for the bacterial protection against environmental stresses [6], [42], [43]. It has been previously shown that *X. axonopodis* pv. citri strains with modified LPS structures are more sensitive to oxidative treatments and UV exposition [14]. In this work, we have observed that *X. axonopodis* pv. citri wild-type was more resistant to oxidants like hydrogen peroxide and MV than Xac*rfb303* and Xac*wzt* (Figure 2). These results indicate that LPS is important for the bacterial survival in oxidative stress conditions, which are usually found during plant-pathogen interactions [44], [45]. In addition, the LPS mutants exhibited an increased sensitivity to SDS compared to the parental strain (Figure 2). It has been proposed that changes in LPS sugar contents could make the bacteria more sensitive to detergents by increasing cell surface hydrophobicity [46]. Our results clearly indicate that Xac*wzt* and Xac*rfb303* are more susceptible to external stresses probably due to their altered LPSs, demonstrating that this molecule functions as a protective structure allowing the bacterial survival in hostile environments.

Several reports showed that bacterial mutants in LPS biosynthesis, including *X. axonopodis* pv. citri, were defective in motility capability [14], [47]. Experiments performed with *Salmonella spp*. associated the presence of an intact LPS molecule with swimming and swarming bacterial motilities [48]. In addition, rough colony appearance, autoagglutination, and loss of motility were correlated to the production of modified LPSs by Gram negative bacteria [49], [50]. Furthermore, the presence of a truncated LPS was related with a decrease or loss of flagella levels [42], [51]. Accordingly, Xac*wzt* exhibited reduced swimming and swarming motilities and lower expression levels of flagellin protein compared to wild-type and Xac*rfb303* strains (Figure 3). However, a study carried out with *Pseudomonas aeruginosa* LPS mutants showed deficiency in swarming motility but not in the assembly of the flagella [52]. In contrast, our results indicate that the presence of a truncated polysaccharide in the O-antigen in the Xac*wzt* strain influences bacterial motilities by altering flagellum biosynthesis.

Most bacteria can attach to solid surfaces and form biofilms, which are defined as matrix-enclosed microbial populations adhering to each other and to surfaces [53], [54]. Recent reports propose that bacterial adhesion and motility are required at initial stages of *X. axonopodis* pv. citri biofilm formation; meanwhile LPS and EPS play important roles in the establishment of a mature biofilm [14], [16]. In our work Xac*wzt* showed higher adherence to abiotic and biotic surfaces but presented neither structured organization nor biofilm formation (Figure 4 and 5). A lot of evidence suggests that LPS is involved in bacterial cell adhesion to both abiotic [55]–[57] and biotic [57], [58] surfaces. Although in many bacteria modification of the LPS structure lead to a reduced adhesion capability to different surfaces [59]–[61], in other species the opposite effect was also observed. It was previously shown that a *Pseudomonas fluorescens* mutant producing a defective O-antigen LPS structure presented an increased adhesion ability to hydrophobic surfaces as a consequence of the higher exposition of the lipid moiety in the surface of the bacterial cell [55]. In addition, Hölzer *et al.* reported a *Salmonella enterica* LPS mutant that showed increased adhesion to animal cell cultures [62]. LPS mutants of *P. aeruginosa* and *Rhizobium leguminosarum* that exhibited an increased adhesion capability were also described [63], [64].

On the other hand, in a study performed by Lindhout *et al.* it was shown that defects in bacterial motility could be explained because changes in the cell-surface due to an altered LPS structure affected bacterial adhesion capability to different surfaces. They proposed that the increase of cell attachment to the agar matrix reduced bacterial motility, without a modification in the flagella biosynthesis [52]. In concordance with Lindhout *et al.* proposal, we observed an increased ability of adhesion of Xac*wzt* to different surfaces along with a reduced motility, although flagella biosynthesis was also altered in our mutant. The increased ability of adhesion of Xac*wzt* could be a consequence of a higher exposure of outer membrane components, such as adhesins and/or an increased hydrophobicity of the cell surface.

Xac*wzt* mutant also exhibited reduced production of EPS compared to wild-type cells (Table 2). This latter observation together with the reduced motility and altered LPS structure of this mutant could explain its impaired ability to form an structured biofilm [14], [16]. On the other hand, Xac*rfb303* mutant presented similar adherence ability and EPS production to *X. axonopodis* pv. citri wild-type and was able to develop a mature biofilm (Figure 5).

Several reports of *Xanthomonas* spp. indicate that the composition of LPS might suffer modifications to avoid the plant recognition [65], [66]. Recently, Li and Wang suggested that the *X. axonopodis* pv. citri LPS biosynthesis could modify its expression at the end of bacterial growth in the colony [16]. Moreover, several studies with animal pathogens demonstrated that LPS structure is modified inside the host in order to prevent defense responses triggered by the host cell [67]. In this work we have studied the expression of *wzt* and *rfb303* genes in XVM2, a medium that mimics the environmental apoplastic space [22]. We observed that the expression of *rfb303* gene, coding a hypothetical core glycosyltransferase, was repressed in this medium (Figure 6). Accordingly, in a previous work Astua-Monge *et al.* using DNA macroarrays found four genes potentially involved in the synthesis of EPS or LPS from *X. axonopodis* pv. citri that were down-regulated in XVM2 medium [68]. We speculate that such effect might occur in wild-type bacteria that are exposed to stresses such as those encountered during plant colonization and disease.

During the interaction with orange plants, Xac*rfb303* produced a faster appearance of water soaking and a more necrotic lesion at final stages of the infection compared to wild-type bacteria. These results were consistent with the differences observed in the growth curves *in planta*, with Xac*rfb303* reaching higher population density than the parental strain. In contrast, host plant inoculation with Xac*wzt* rendered a less aggressive phenotype with minor damage of the plant tissue respect to *X. axonopodis* pv. citri wild-type. Accordingly, the number of bacterial cells recovered from the leaf apoplast was lower for this strain (Figure 7). Mutants with defective LPS showing reduced virulence have been isolated from all the major genera of bacterial pathogens [6], [69]–[74]. The higher sensitivity to oxidants observed in Xac*wzt* mutant could be responsible for the reduced symptoms severity in the plant and the decrease of the number of viable cells recovered from the plant tissue. Cell membrane injuries produced in leaves by the different bacterial inoculations were also in concordance with the phenotypes observed (Table 3). Our results indicate that the structural modification of *X. axonopodis* pv. citri LPS through *wzt* mutation affects several physiological features of this bacterium, reducing the pathogenicity of *X. axonopodis* pv. citri in host plants. *In silico* analysis of different *Xanthomonas* genomes revealed that this gene is present in a complete version in *X. axonopodis* pv. citri but have lost the C-terminus region in other related citrus canker species, which was correlated with their different levels of virulence. Specifically, Moreira *et al.* reported that *Xanthomonas fuscans* subsp. *aurantifolii* type B and *Xanthomonas fuscans* subsp. *aurantifolii* type C, two canker producing strains that have a truncated *wzt* gene, produced a less virulent lesion in citrus plants with a minor water soaking production compared to *X. axonopodis* pv. citri in concordance with the phenotype observed with Xac*wzt* mutant [75]. On the other hand, taking into account the phenotype produced by Xac*rfb303* during the pathogenesis process in the host plant and the expression of *rfb303* gene in XVM2 medium we suggest that probably *X. axonopodis* pv. citri could induce LPS structural modifications *in planta* in order to produce a better condition for the colonization or establishment in the host plant tissue. This assumption could be consistent with Silipo *et al.* findings where a *Xanthomonas campestris* pv. campestris mutant defective in core completion showed that modifications in the acylation and phosphorylation patterns of its lipid A influences plant responses [76].

In contrast with the role of the LPS in promoting plant disease by acting as a barrier against host compounds, LPS has a role in the induction of plant innate immunity, acting as a PAMP, an essential structure present in pathogenic and nonpathogenic bacteria [13]. The role of *X. axonopodis* pv. citri LPS as a PAMP was demonstrated in a previous work where LPS isolated from Xac*wzt* was unable to induce callose deposition, stomata closure and ROS production in orange leaves compared with *X. axonopodis* pv. citri wild-type [20]. In addition, it has been reported that acting as a PAMP, LPS activates signaling mechanisms and cellular responses in plants, including the induction of mitogen-activated protein kinase (MAPK) cascades [77], redox enzymatic systems (GST and Prxs) [78] and defense-related genes (PRs) [79]. We have previously demonstrated that LPS isolated from *X. axonopodis* pv. citri wild-type is capable to induce accumulation of several transcripts related to basal defense like *PR-1* and *MKK4* [20]. In the present work we observed that inoculation of orange leaves with *X. axonopodis* pv. citri mutant strains produced a lower increase in the expression levels of *PR-1*, *MKK4*, *PrxA* and *GST* genes compared to *X. axonopodis* pv. citri wild-type. This reduction in the expression levels was more pronounced for the Xac*rfb303* mutant, in spite of the higher growth of this mutant *in planta* (Figure 8). These results indicate that the alteration of *X. axonopodis* pv. citri LPS structure influence the basal response in orange plants corroborating its role as a PAMP. Additionally, the reduced basal response generated by Xac*rfb303* could be associated with the improved growth of this strain inside host tissues.

The most common defense response exhibited by plants against pathogenic microorganisms is the non host response generally characterized by ROS accumulation, localized hypersensitive response and cell death restricting the pathogen growth [80], [81]. Plants non host resistance is activated by the recognition of PAMPs molecules, like LPS [82], [83]. It is well known that *X. axonopodis* pv. citri induces HR in several non host plants. Since citrus plants resistant to *X. axonopodis* pv. citri infection have not been found, non host plants were used to characterize basal defenses induced by this bacterium [21], [84]. In this work we showed that *X. axonopodis* pv. citri wild-type produced a typical HR lesion with a necrotic area on tobacco leaves. However, LPS mutant strains were not able to generate this kind of lesion on tobacco plants (Figure 9). Therefore, the modifications in the LPS molecule composition affected the development of HR on the non host plant suggesting a role of the LPS in this response.

Pretreatment of pepper leaves with LPS prior to bacterial inoculation has been shown to reduce subsequent symptom development caused by *Xanthomonas campestris* pv. vesicatoria [85]. The prevention of HR reflects an increased resistance of the plant tissue to bacterial attack. In this context, we have demonstrated that LPS from *X. axonopodis* pv. citri wild-type inhibited the appearance of HR symptoms when tobacco leaves where subsequently inoculated with living bacteria, corroborating that *X. axonopodis* pv. citri LPS activates the plant defense in tobacco (Figure 9). This result is consistent with the previously reported induction of peroxide levels in tobacco leaves inoculated with *X. axonopodis* pv. citri wild-type LPS, which is characteristic of plant basal defense [20].

In this work we modified two different regions of the *X. axonopodis* pv. citri LPS, the core region and the O-antigen. Our data constitute the first description of *X. axonopodis* pv. citri mutant in the core region. These LPS alterations affect components of the cell surface that influence several bacterial physiological features and the interaction with host and non host plants. In conclusion, we suggest that *X. axonopodis* pv. citri LPS not only acts as a virulence factor but also induces plant defense responses during the compatible interaction with orange plants. Additionally, we suggest that the different components of the LPS would have different contributions to the dual role of this macromolecule during the plant colonization.

## Supporting Information

Figure S1 **Protein expression profile of bacteria from swarming plates.** Equal amounts of protein extracts of bacteria harvested from the center and the border of swarming plates were resolved by SDS-PAGE and analyzed by staining with a 0.1% (w v^−1^) Coomassie Brilliant Blue R-250 solution.
(TIF)

Figure S2 **Exopolysaccharide production.** Mucoid aspect of Xac colonies provided by xanthan production was analyzed by growing Xac wild-type, Xac*wzt*, Xac*rfb303*, Xac*Cwzt* and Xac*Crfb303* on SB solid media, at 28°C during 48 h.
(TIF)
