# Supplementary figures and images for: Modifications of Xanthomonas axonopodis pv. citri Lipopolysaccharide Affect the Basal Response and the Virulence Process during Citrus Canker
Source: PLoS One. 2012 Jul 6;7(7):e40051. doi: 10.1371/journal.pone.0040051 (PMC3391215; doi:10.1371/journal.pone.0040051)

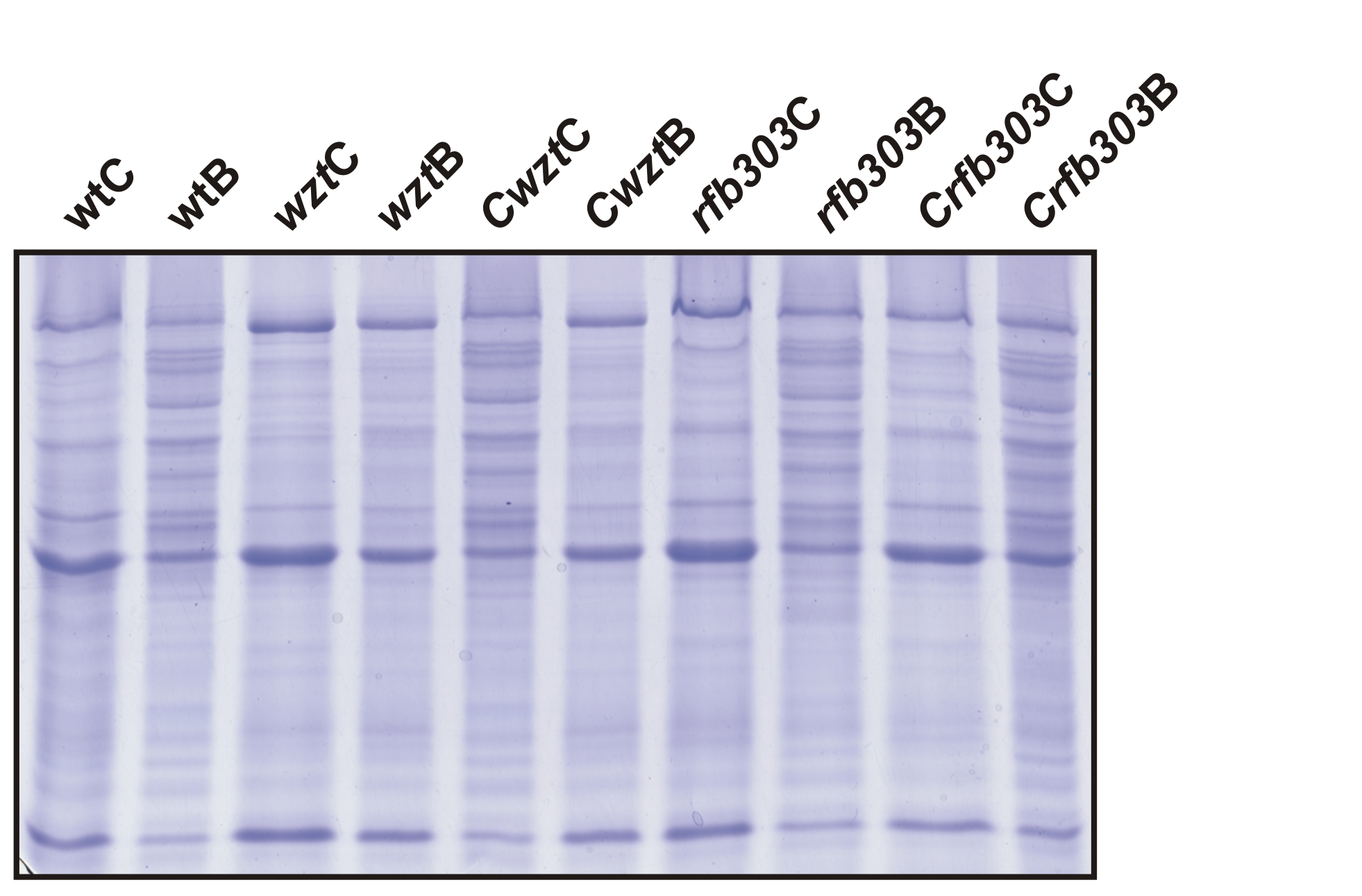

Supplement: Figure S1 — Protein expression profile of bacteria from swarming plates. Equal amounts of protein extracts of bacteria harvested from the center and the border of swarming plates were resolved by SDS-PAGE and analyzed by staining with a 0.1% (w v−1) Coomassie Brilliant Blue R-250 solution. (TIF) [file pone.0040051.s001.tif]

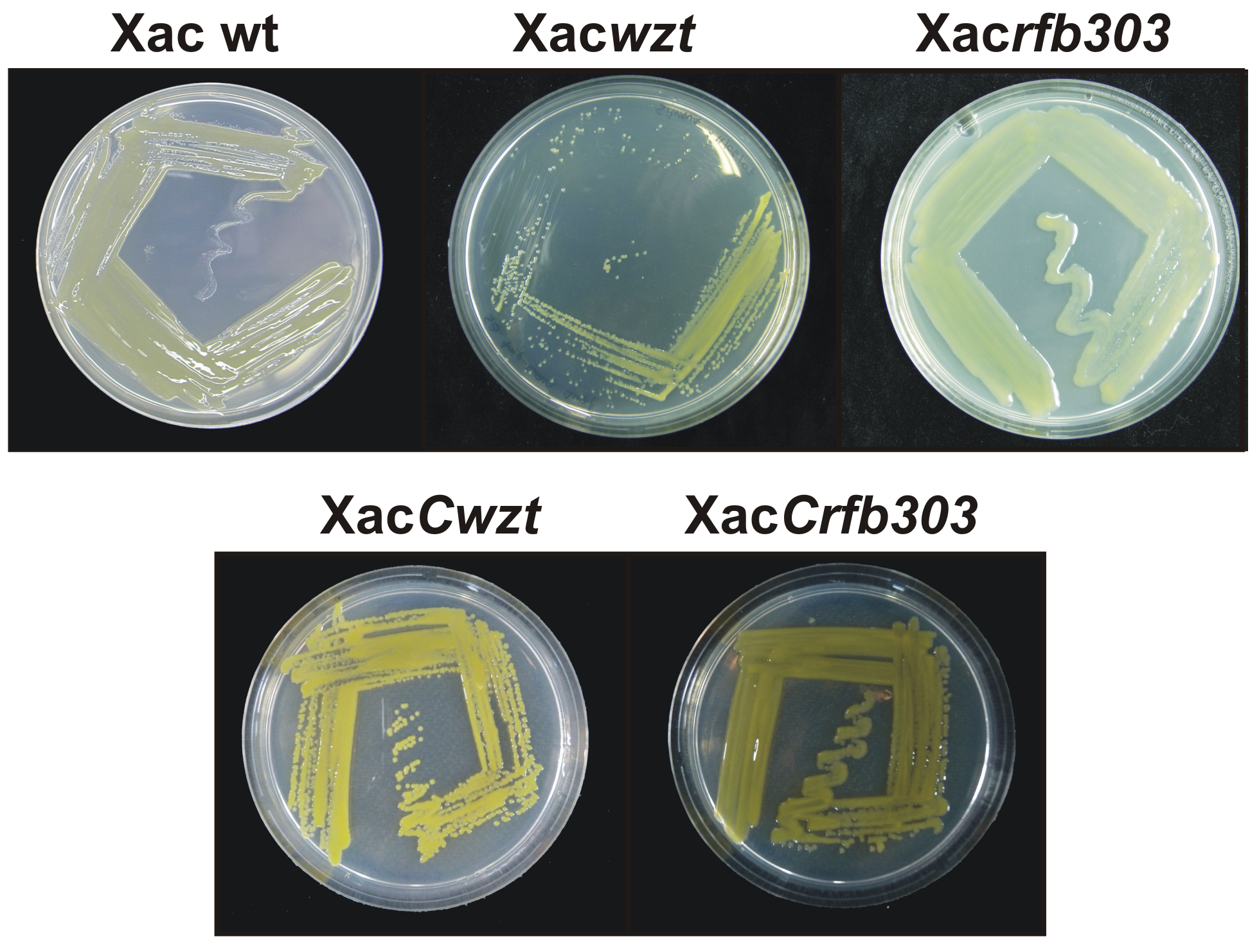

Supplement: Figure S2 — Exopolysaccharide production. Mucoid aspect of Xac colonies provided by xanthan production was analyzed by growing Xac wild-type, Xacwzt, Xacrfb303, XacCwzt and XacCrfb303 on SB solid media, at 28°C during 48 h. (TIF) [file pone.0040051.s002.tif]
